# Interaction of Organic Semiconductors and Graphene Materials in the Source-Drain Channel of Field-Effect Transistors

**DOI:** 10.3390/bios15090622

**Published:** 2025-09-19

**Authors:** Eugen Chiriac, Bianca Adiaconita, Tiberiu Burinaru, Catalin Marculescu, Marius Stoian, Catalin Parvulescu, Marioara Avram

**Affiliations:** National Institute for Research and Development in Microtechnologies—IMT Bucharest, 126A Erou Iancu Nicolae, 077190 Voluntari, Ilfov, Romania; bianca.adiaconita@imt.ro (B.A.); tiberiu.burinaru@imt.ro (T.B.); catalin.marculescu@imt.ro (C.M.); marius.stoian@imt.ro (M.S.); catalin.parvulescu@imt.ro (C.P.)

**Keywords:** graphene-based FETs, TTF, HAT-CN, π–π interactions, charge transfer, SEM, Raman spectroscopy, FTIR, molecular electronics, biosensors

## Abstract

This study investigates the interfacial interactions between two organic semiconductors (tetrathiafulvalene (TTF) and hexaazatriphenylene-hexacarbonitrile (HAT-CN)) and graphene-based materials (nanocrystalline graphite and vertically aligned graphene) used in Field-Effect Transistors (FETs). The interaction mechanisms, including π–π stacking, charge transfer, and dipole–dipole interactions, were explored through SEM imaging, Raman and FTIR spectroscopy, and FET transfer characteristics. Spectroscopic data confirmed strong π–π and charge-transfer interactions, with distinct modifications in graphene structural and electronic features. Electrical measurements revealed significant modulation of channel conductivity, confirming effective surface functionalization. These findings provide a framework for engineering high-performance organic/graphene hybrid interfaces in electronic devices and biosensors. Importantly, the results demonstrate that molecular design and interfacial control at the nanoscale can be strategically used to modulate charge transport in graphene-based FETs. This approach opens new pathways for developing tunable, molecule-specific biosensors and nanoelectronic platforms with enhanced sensitivity and selectivity.

## 1. Introduction

Diverse types of small biomolecules (e.g., glucose, dopamine, lactate, etc.) and macromolecules (e.g., DNA, RNA, proteins, lipids, etc.) with a wide range of functions can be used for early detection of disease using biosensors. By combining a biological component (e.g., enzymes, antibodies, ssDNA/RNA probes, aptamers, etc.) with a physicochemical detector, biosensors can convert a biochemical signal into a detectable and quantifiable electrical signal. In recent years there has been a high increase in wearable gadgets and wireless electronic health monitoring systems that can be used as digital biomedical tools in digital hospitals, e-health, and telemedicine [1]. There is an estimated growth of wearable gadgets from 300 million in 2016 to 1 billion by 2024 [1]. All these systems, platforms, and devices integrate and use several types of sensors like electrochemical sensors [2], field-effect transistors [3], optical biosensors [4], piezoelectric biosensors [5], and capacitive, resistive, amperometric, and potentiometric sensors [6].

To increase the sensitivity and decrease the size of the biosensor, new nanomaterials like nanoparticles, carbon nanotubes (CNTs), nanowires, quantum dots, graphene, and 3D carbon nanomaterials like vertical graphene and nanocrystalline graphite have been used to enhance the functionality of biosensors, widening their applicability and utility in the health sector [7,8,9,10,11,12,13,14,15]. However, the production of these nanomaterials requires expensive equipment and specialized facilities and personnel, which increases the production cost of the final biosensor.

To overcome conventional semiconductor materials, a new class of organic semiconductors is being researched with promising applications for field effect transistors, solar cells, and light-emitting diodes [16]. In recent years, research has been focused on developing biosensors based on organic electrochemical transistors (OECTs) and organic field-effect transistors (OFETs) for wearable and implantable biomarker detection [17]. Due to their biocompatibility, low operating voltage, and signal amplification capability, they can be used for pH, ions, molecules, and biomarker sensing. Especially organic field-effect transistors (OFETs), due to their low cost, mechanical flexibility, and ease of bio functionality, offer a promising alternative for biosensing [18]. The general structure of an OFET comprises gate, source, and drain electrodes with a transistor channel made of organic semiconductors (OSCs) or organic mixed ion–electronic conductors (OMIECs) and a dielectric. An electrolyte-gated organic field-effect transistor (EGOFET) has an organic semi-conducting layer in contact with an electrolyte and the gate on top of the electrolyte solution sensing area [19]. Depending on the type of the OFET, there are three biosensing mechanisms that can be used: gate functionalization, channel interface functionalization, and electrolyte functionalization. Organic semiconductors used in OFETs that donate electrons, involving a high HOMO (highest occupied molecular orbital) level are well suited for p-type semiconductors, while electron-accepting organic semiconductors with a low LUMO level are well suited for n-type semiconductors [20]. The most common used organic semiconductor is P3HT (Poly(3-hexylthiophene)), which showed hole mobilities higher than 0.1 cm^2^/Vs and very high on/off ratios (>10^5^) [21]. Another OSC is represented by pBTTT (Poly(2,5-bis(3-alkylthiophen-2-yl)thieno[3,2-b]-thiophene)), which possess hole mobility up to 0.1–10 cm^2^/Vs [22]. Pentacene is another OSC commonly used, with a charge mobility of about 0.38 cm^2^/Vs [23].

Since the mobility of the OFET is dictated by the intermolecular π-orbital overlapping, and efficient energy-level alignment is essential to increase the sensitivity of organic electronic devices, new organic semiconductor materials have been researched. Two promising examples are represented by 1,4,5,8,9,11-hexaazatriphenylene hexacarbonitrile (HAT-CN) and tetrathiafulvalene (TTF). To increase sensitivity for high-performance organic electronic devices, the energy barrier between the Fermi level of the anode and the HOMO level must be reduced. HAT-CN is used as a hole injection layer (HIL) of an adjacent p-type organic semiconductor. HAT-CN’s electronic structure and charge transport via π-orbital stacking differ according to the molecular orientation [16,24]. TTF molecules were first synthesized in the 1970s, and since then they have been studied for their strong electron-donor abilities [25]. TTF molecules possess strong electron-donor abilities with a high HOMO level due to the sulfur atoms at its core. In 1995, Mulchandani et al. developed a TTF based, amperometric biosensor with a carbon paste electrode for the quantification of L-lactate concentrations in milk and yogurt with an LOD of 56 µM [26]. Since then, TTF has been extensively studied by the international research community. Because different molecules can be further attached to the TTF core, the material has been used in multiple application electronics, optoelectronics, chemistry, magnetism, or in molecular devices [27]. Recent studies show that, just as HAT-CN, TTF can be used to enhance the electrical properties of graphene, which is already considered an excellent material with exceptional electric and mechanical properties used in energy and electronics, bio-medical, and environmental fields [28]. Nair et al. functionalized epitaxial graphene with a TTF derivative functionalized with four alkylthio substitutional groups, which showed an increase in the electrical conduction of carrier mobility up to 6–20 cm^2^/Vs. The authors explain that the alkylthio chains force the molecules to adopt an edge-on conformation, which reduces the distance separation between TTF cores lower than 3.8 Å, leading to a full π–π overlapping and increased molecular alignment between the graphene and TTF layers, which in turn leads to increased carrier mobility [29]. Chen et al. used a HAT-CN/Graphene sandwich composite, by layer-by-layer self-assembly of HAT-CN on the surface of GO, for the fabrication of lithium-ion batteries (LIBs). The HAT-CN/Graphene cathodes exhibit superior reversal capacity, cyclic stability, and rate capability, which is much better compared to pure HAT-CN cathodes [30]. Parfenov et al. developed a HAT-CN-based OFET gas sensor with a charge carrier mobility of 10^−4^ cm^2^/Vs. They achieved a limit of detection (LOD) of ≈10 ppb or even lower [31]. Huang et al. developed a hydrogen peroxide (H_2_O_2_) biosensor for reduced nicotinamide adenine dinucleotide (NADH) detection. They fabricated a biosensor based on a glass carbon electrode modified with CNTs/TTF/HRP, a platinum (Pt) wire counter electrode, and an Ag/AgCl reference electrode. Using EIS measurements, they achieved a linear response range from 10 μM to 790 μM, with a sensitivity of 4.76 μA/mM and an LOD of 1.53 μM [32]. There are currently no studies that describe the interaction between TTF and HAT-CN with vertical graphene (VG) and nanocrystalline graphite (NCG) and the influence that they possess on the electrical properties of these new types of 3D carbonic materials. Also, there are no sensors or biosensors that use VG and NCG together with TTF or HAT-CN [33,34].

In the current work we propose to explore and understand the interaction between TTF and HAT-CN as organic semiconductors with 3D carbonic nanomaterials like vertical graphene (VG) and nanocrystalline graphite (NCG) and their influence on the electrical properties of two types of graphene-derived nanomaterials.

## 2. Materials and Methods

### 2.1. Microfabrication of Field-Effect Transistors Based on 3D Graphene (VG and NCG)

The fabrication process of the FET structures involves the deposition of a graphene layer (NCG and VG) on an oxidized silicon wafer, followed by the definition of metal contacts for the source, drain, front gate, and back gate. In these processes, we use five photolithographic masks: M1–Graphene (NCG and VG); M2–Source-Drain; M3–Front Gate (Al_2_O_3_); M4–Passivation and Oxidation; M5–Back Gate.

**Figure 1:**
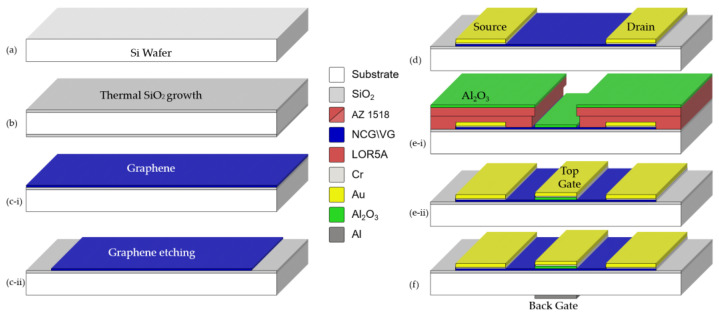
Schematic presentation of the technology flow for NCG/FET fabrication: (**a**) Si wafer used as substrate for device fabrication; (**b**) SiO_2_ growth; (**c-i**) PECVD deposition of nano-crystalline graphite; (**c-ii**) graphene etching; (**d**) fabrication of Cr/Au source-drain electrodes by lift-off; (**e-i**) ALD deposition of Al_2_O_3_ layer; (**e-ii**) fabrication of Cr/Au front gate by lift-off method; (**f**) fabrication of aluminum back gate by lift-off method.

The complete technological workflow is illustrated in Figure 1 and described in detail below:

(a) The process began with a <100> -oriented, p-type silicon wafer, cleaned in hot Piranha solution (H_2_SO_4_ : H_2_O_2_ = 3:1) at ~120 °C for 30 min to remove organic contaminants. The resulting chemical oxide was removed by immersion in diluted HF (1–2%) for 30 s, followed by deionized water rinsing and nitrogen drying.

(b) A wet thermal oxidation process was performed at 900 °C for 340 min, resulting in ~500 nm of SiO_2_ grown on both sides of the wafer. The backside oxide was then removed by buffered HF etching to allow substrate contact, followed by DI water rinsing and nitrogen drying.

(c) Mask M1: NCG and VG were synthesized by PECVD on separate oxidized Si wafers: NCG at 900 °C (CH_4_: 60 sccm, H_2_: 75 sccm), and VG at 750 °C (CH_4_: 10 sccm, Ar: 190 sccm). Active areas were defined via photolithography (Mask M1), followed by oxygen plasma etching and photoresist removal. The wafers were then rinsed and dried under nitrogen.

(d) Mask M2: The source and drain electrodes were defined using Mask M2 and a bilayer lift-off process. LOR 5A and AZ 1518 photoresists were sequentially spin-coated onto the oxidized Si wafer and soft-baked then exposed to UV light through the source/drain photomask. After development, a Cr/Au bilayer (20 nm/200 nm) was deposited by thermal evaporation. Lift-off was carried out in acetone with mild ultrasonication. The metal electrodes partially overlap the graphene channel to form electrical contacts, while their extended portions serve as probing pads for subsequent characterization.

(e) Mask M3: The front gate electrode was fabricated using a bilayer lift-off process (LOR 5A/AZ 1518) combined with localized dielectric deposition. After photolithographic definition of the gate area using Mask M3, a 20 nm thick aluminum oxide (Al_2_O_3_) layer was deposited by atomic layer deposition (ALD) at 120 °C. This was immediately followed by Cr (30 nm) and Au (335 nm) metal deposition by thermal evaporation. Lift-off was performed in solvent with mild ultrasonication, resulting in the front gate electrode precisely aligned over the dielectric. This integrated process minimizes potential misalignments between the oxide and the metal layer.

(f) Mask M4: The back gate electrode was fabricated on the rear side of the wafer using Mask M5 and a bilayer lift-off process (LOR 5A/AZ 1518). After photolithographic patterning, a 300 nm thick aluminum layer was deposited by thermal evaporation. Lift-off was performed in solvent with ultrasonication to define the metal contact. The aluminum was deposited directly onto the native oxide present on the silicon surface, without prior oxide removal.

In the final step, the wafer was diced into individual chips containing the fabricated FET structures. The dicing process was performed using a precision diamond blade wafer saw. Each chip includes a single transistor with its associated contact pads, allowing for individual electrical characterization.

### 2.2. Surface Activation Using Organosulfured Compounds Electron Donor (TTF)

**Figure 2:**
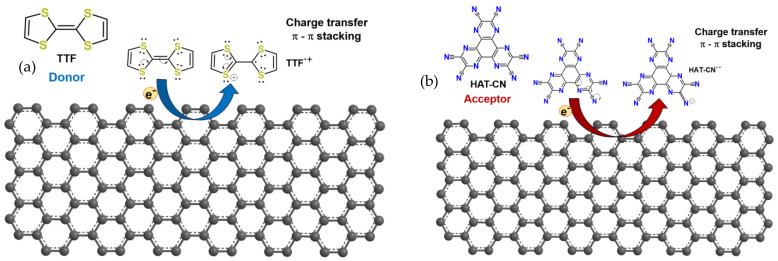
(**a**) Graphene-TTF interaction; (**b**) graphene interaction with HAT-CN.

Surface activation using electron-donating organosulfur compounds (TTF) can modify the Fermi levels of graphene-based nanomaterials (VG, NCG), thereby influencing the electrical conductivity and transport characteristics of the FET, as depicted in Figure 2a. Tetrathiafulvalene (TTF), with the formula (C_3_H_2_S_2_)_2_, is an organosulfur compound that contributes to the development of molecular electronics. TTF has a planar structure and is derived from the hydrocarbon fulvalene (C_5_H_4_)_2_ by replacing the four CH groups with sulfur atoms. It is also an aromatic compound, exhibiting delocalized electrons in its rings.

TTF has a strong electron-donating ability due to its high-energy highest occupied molecular orbital (HOMO) level, measured at 4.73 eV, which is close to the work function of common metal electrodes. This results in a low-energy barrier for charge injection from the metal electrodes into the active layer. The bandgap of TTF is approximately 1.1 eV. The HOMO level of TTF is crucial for understanding its electronic properties. TTF exhibits a prominent HOMO level due to its extended conjugated π system, consisting of alternating sulfur and carbon atoms. This low-energy HOMO makes TTF a suitable candidate for electron donor materials in organic electronics and a key building block in molecular electronics thanks to its effective electron donation capability. These properties contribute to the high-performance organic field-effect transistors (OFETs) based on TTF nanostructures.

TTF was purchased from Sigma Aldrich (St. Louis, MO, USA) and used with no further purification. The organic compound was dissolved in dichloromethane, reaching a concentration of 4 mg/mL. A droplet of the TTF solution (4 µL) was cast onto the substrate and allowed to evaporate in a chamber filled with dichloromethane vapor at room temperature. The substrates used consisted of source-drain channels made from VG or CVD-grown NCG on SiO_2_ substrates. Prior to the drop casting process, all substrates were cleaned with isopropyl alcohol.

### 2.3. Surface Activation Using Organonitrogen Compounds Electron Acceptor (HAT-CN)

Surface activation using electron-accepting compounds (HAT-CN) can stabilize the Fermi levels of graphene, thereby influencing the switching behavior of the FET, as presented in Figure 2b. Hexaazatriphenylene-hexacarbonitrile (C_18_N_12_ or HAT-CN) is a polycyclic aromatic hydrocarbon composed of nitrogen and carbon atoms arranged in a hexagonal ring structure that is used in organic electronics, particularly in the field of organic semiconductors. The bandgap of HAT-CN is approximately 2.3 eV. HAT-CN exhibits excellent electron transport properties, making it a highly efficient p-type semiconductor material.

Its unique molecular structure and electron-deficient nature make it suitable for applications such as organic field-effect transistors (OFETs), organic light-emitting diodes (OLEDs), and organic photovoltaic cells (OPVs). Researchers are actively exploring HAT-CN and its derivatives to enhance device performance, stability, and compatibility across a range of organic electronic applications. These properties make it a promising candidate for next-generation electronic devices.

The redox properties of HAT-CN make it a versatile material for various electronic and electrochemical applications, offering opportunities for tailored device design and functionality. HAT-CN displays interesting redox behavior and is a strong electron acceptor due to its unique molecular structure and the presence of numerous nitrogen atoms. These properties enable HAT-CN to act as an electron transporter in devices such as OFETs and OPVs.

HAT-CN can undergo reduction reactions, in which it gains electrons and forms reduced species. This process is often exploited in electrochemical studies and can be used to modulate the electronic properties of HAT-CN-based materials. It can also undergo oxidation reactions, in which it loses electrons and forms oxidized species.

HAT-CN was purchased from Sigma Aldrich (St. Louis, MO, USA) and used with no further purification. The organic compound was dissolved in acetone, reaching a concentration of 6 mg/mL. In a similar manner for the TTF deposition, a droplet of the HAT-CN solution (2 µL) was casted onto the substrate and allowed to evaporate in a chamber filled with acetone vapor at room temperature.

### 2.4. Material and Device Characterization

Morphological characterization was performed using scanning electron microscopy (SEM) to investigate the surface structure and layer formation of the deposited materials. For this purpose, a Field Emission Gun Scanning Electron Microscope (FEG-SEM)—Nova NanoSEM 630 (FEI Company, Hillsboro, OR, USA)—was employed.

Raman spectroscopy measurements on the samples were carried out by a high-resolution Scanning Near-Field Optical Microscope fitted with the Raman Module Witec Alpha 300S (Witec, Ulm, Germany), using a laser with a 532 nm wavelength (laser excitation energy of 2.33 eV).

FTIR spectra of all the samples were acquired using the VERTEX 80/80v FT-IR spectrometer, from Bruker Optics (Ettlingen, Germany), equipped with a reflectivity module, at an incidence angle of 45 degrees, in the 4000–400 cm^−1^ spectral domain, and with a resolution of 4 cm^−1^.

Electrical measurements were performed using a Semiconductor Characterization System (DC) with Wafer Probing Station—4200-SCS/C/Keithley Easyprobe EP6/Suss MicroTec (Keithley Instruments, Beaverton, OR, USA; Suss MicroTec, Germany).

Regarding the device characterization, in this work, several individual GFET structures (NCG and VG-based), obtained on the same silicon wafer, were each functionalized with specific semiconductor molecules (HAT-CN and TTF) to evidence the repeatability of the deposition method and of the results. In this way, the individual structures were marked on figures with the corresponding labels.

## 3. Results and Discussions

### 3.1. Morphological Characterization of FET Devices Functionalized with Organic Semiconductor Molecules

**Figure 3:**
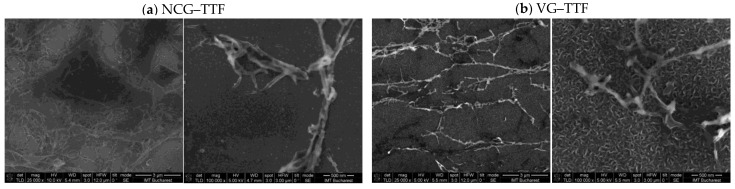
Scanning electron microscopy images of (**a**) NCG–TTF and (**b**) VG–TTF.

The surface morphology of the hybrid materials obtained by functionalizing graphene with tetrathiafulvalene (TTF) was investigated using scanning electron microscopy (SEM), as shown in Figure 3. Two types of graphene structures were analyzed: nanocrystalline graphite (NCG) and vertically oriented graphene (VG), each combined with TTF.

In the case of the NCG–TTF sample, SEM micrographs reveal a granular, disordered morphology, with irregular TTF aggregates distributed across the amorphous graphene surface. The coating appears patchy and non-uniform, suggesting weak and uncontrolled π–π interactions between the TTF molecules and the disordered carbon network. This morphology may lead to inefficient charge-transfer pathways, which could negatively impact the electrochemical performance of the material.

In contrast, the VG–TTF sample exhibits a markedly different structure. The vertically aligned graphene architecture presents a porous, high-surface-area morphology, with graphene sheets oriented perpendicularly to the substrate. TTF appears adsorbed in a more defined and homogenous manner on the exposed edges and surfaces of the vertical graphene walls. This well-organized configuration promotes stronger and more effective π–π stacking interactions, which are likely to enhance charge-transfer efficiency and make the VG–TTF system a more promising candidate for applications in electrochemical sensors or organic electronic devices.

Overall, SEM analysis highlights the critical influence of graphene architecture on the spatial distribution and interaction mode of TTF, directly impacting the functional potential of the resulting hybrid material.

**Figure 4:**
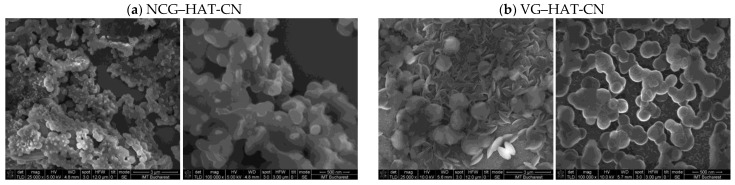
Scanning electron microscopy images of (**a**) NCG–HAT-CN and (**b**) VG–HAT-CN.

The morphology of graphene-based hybrid materials functionalized with 1,4,5,8,9,12-hexaazatriphenylene-hexacarbonitrile (HAT-CN) was examined via scanning electron microscopy (SEM), as shown in Figure 4. Two structural forms of graphene were employed: nanocrystalline graphite (NCG) and vertically aligned graphene (VG), each drop-casted with HAT-CN.

SEM images of the NCG–HAT-CN sample display a surface densely covered with spherical aggregates, indicating extensive HAT-CN self-assembly on the disordered graphene substrate. The globular morphology suggests strong intermolecular interactions between HAT-CN molecules, which dominate over interactions with substrate due to the lack of structural ordering in NCG. The π–π stacking with the disordered graphene appears inefficient or spatially limited, restricting effective charge transfer between the donor substrate and the strong electron acceptor (HAT-CN).

In contrast, the VG–HAT-CN sample reveals fewer but significantly larger and more defined HAT-CN aggregates, some of which appear as flattened or layered particles on the exposed graphene sheets. The vertical orientation of VG provides accessible edge planes and enhanced surface area, which facilitates more favorable π–π interactions and a better-defined charge-transfer interface. Additionally, the smoother background textures in the VG–HAT-CN images suggest less uncontrolled self-assembly and a more uniform distribution of HAT-CN across the conductive substrate.

These morphological differences emphasize the crucial role of graphene architecture in directing the assembly behavior of HAT-CN and the formation of efficient charge-transfer complexes. While NCG tends to promote uncontrolled aggregation of HAT-CN, VG supports a more ordered and electronically favorable hybrid structure.

A comparative evaluation of the SEM images presented in Figure 3 and Figure 4 reveals distinct morphological behaviors depending on the type of molecular dopant (TTF vs. HAT-CN) and the architecture of the graphene substrate (NCG vs. VG).

In both systems, the choice of graphene substrate plays a significant role in dictating the nature of the hybrid interface. For TTF, a planar, electron-rich π-donor, and interactions with NCG lead to a disordered and uneven surface distribution, characterized by small, irregular aggregates. In contrast, TTF on VG exhibits better-defined particle deposition and a more controlled morphology, attributed to the higher surface area and exposed edge planes of the vertical graphene structure. This indicates more favorable π–π stacking and improved charge-transfer interactions with VG compared to NCG.

On the other hand, HAT-CN, a strong electron acceptor with multiple cyano groups, tends to self-assemble into spherical aggregates regardless of the substrate. However, the morphology of HAT-CN on NCG shows dense, globular packing with limited substrate integration, suggesting dominant intermolecular interactions and weak coupling with the disordered graphene. In contrast, HAT-CN on VG forms fewer but larger and more organized aggregates, with indications of interfacial flattening and integration into the vertical graphene structure. This suggests that VG not only enables better π–π stacking but also imposes structural directionality on the self-assembly of HAT-CN.

Overall, VG supports more uniform, well-integrated, and electronically favorable hybrid architectures with both TTF and HAT-CN, while NCG promotes disordered or excessive aggregation, especially in the case of HAT-CN. These differences highlight the importance of graphene morphology in tuning molecular organization and charge-transfer efficiency in donor–acceptor hybrid materials.

### 3.2. Raman Characterization of FET Devices Functionalized with Organic Semiconductor Molecules

Raman spectroscopy is a valuable technique for studying molecular vibrations, rotational modes, and other excitations within a system. While it does not measure binding energy directly, it can contribute indirectly to its determination in the following ways: (i) Identification of vibrational modes: By analyzing the vibrational modes observed in the Raman spectrum, information can be obtained about the forces acting on atoms within a molecule. These forces can be correlated with binding energies through theoretical methods and quantum chemical calculations; (ii) Analysis of molecular structure: Changes in Raman vibrational frequencies can reflect changes in molecular structure, which in turn can be related to modifications in binding energy. For example, strengthening chemical bonds within a molecule will increase the corresponding vibrational frequencies.

#### 3.2.1. Raman Spectrum Interpretation of TTF

**Figure 5:**
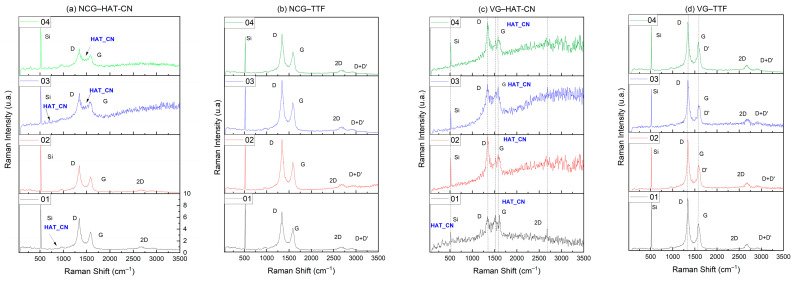
Raman spectra of TTF and HAT-CN on VG and NCG layers grown on SiO_2_ substrate: (**a**) NCG–HAT-CN; (**b**) NCG–TTF; (**c**) VG–HAT-CN; (**d**) VG–TTF. The labels 1, 2, 3, and 4 refer to individual GFET structures (NCG and VG) obtained on the same Si wafer but functionalized with the specific molecules (TTF and HAT-CN).

The TTF structure includes two thiolate rings (S-C=C-S), so its molecular symmetry influences the Raman activity of the vibrational modes, illustrated in Figure 5. The Raman vibration modes of TTF can be categorized as stretching and bending vibrations of the bonds in the molecule: stretching modes C=C in the ~1400–1600 cm^−1^ region and C-S in the ~600–800 cm^−1^ region; bending modes CH in the ~1000–1300 cm^−1^ region and twisting and bending modes of the S-C=C-S rings below ~500 cm^−1^. TTF specific vibrational modes appear in the Raman spectra combined with those of the NCG or VG substrate, indicating the presence of TTF on the surface of the carbonaceous materials (VG and NCG). In this case, the D band (~1350 cm^−1^), G band (~1580 cm^−1^), and 2D band (~2700 cm^−1^) of the carbonaceous material undergo a change in intensity due to the presence of TTF. The molecular symmetry of TTF influences Raman activity on VG and NCG through several mechanisms, which depend on the interaction between TTF and the carbon substrate, the selection of Raman modes, and charge transfer effects.

The TTF has a planar symmetry and belongs to the D2h symmetry group, which means that its vibrational modes are well defined and can be classified as Raman active. On VG and NCG, interactions can induce changes in the effective symmetry by distortions of the molecule or by interactions with substrate defects. On VG, which has a three-dimensional surface with edges and defects, TTF molecules can preferentially adsorb, which can induce a slight distortion of the symmetry of the molecule and the activation of Raman modes that are forbidden in the free symmetry of TTF. In addition, π–π interactions with the graphene walls can lead to changes in the vibrational frequencies. NCG, being a more uniform nanoscale material, allows the TTF-NCG interaction to be more dependent on the density of the electronic states of the nanocrystalline graphite. If the NCG has a non-uniform crystallite size distribution, this may affect the activation of Raman modes by local plasmon effects and charge transfer.

Electron transfer between TTF and VG or NCG leads to changes in the intensity and position of the G and 2D bands of the carbon substrate, as well as TTF modes. VG, having a higher defect density and sharp edges, can facilitate stronger charge transfer, leading to amplification of certain TTF Raman modes by resonance effects. NCG, having a more homogeneous π-band distribution, provides a more stable and predictable interaction with the TTF, leading to a Raman spectrum less affected by variations in charge transfer.

On VGs, thiol groups of the TTF molecules favor anchoring on the reactive defect sites found in the carbon network, which leads to changes in the polarizability of the molecule and, consequently, on the Raman activity. On NCG, the thiol–carbon network interaction is weaker, leading to a Raman spectrum closer to that of the free molecule.

#### 3.2.2. Raman Spectrum Interpretation of HAT-CN

The Raman spectrum of HAT-CN is dominated by stretching modes of the C≡N and C=C/C=N bonds in the aromatic nucleus, with specific bands ~2200–2300 cm^−1^. In the frequency range ~1500–1650 cm^−1^, the stretching modes of C=C and C=N bonds are found, and between ~1100 and 1300 cm^−1^, the bands associated with the stretching of C-N single bonds are found. At low frequency ~400–600 cm^−1^, the bands in this region are related to C≡N bond bending modes and other torsion modes of the aromatic core structure. The specific HAT-CN vibrational modes appear in the Raman spectra and in this case are combined with those of the NCG or VG substrate, indicating the presence of HAT-CN on the VG and NCG surface by a change in the intensity of the carbonaceous materials. HAT-CN is a planar molecule with a rigid structure and an extended π-electron system, which makes it susceptible to π–π interactions and charge transfer when adsorbed on carbonaceous surfaces such as VG and NCG.

Both VG and NCG have π-electron-rich surfaces, and HAT-CN, being an aromatic molecule, can interact strongly with these substrates. Due to its three-dimensional surface area and high edge density, VG provides HAT-CNs with a medium with numerous adsorptions via π–π interactions and additional interactions with edge defects. This leads to a change in the position and intensity of the Raman bands of the HAT-CN by perturbing the electronic and vibrational symmetry. NCG, being more uniform on the nanoscale and having a structure close to that of graphite, favors weaker but more ordered interactions with HAT-CN.

HAT-CN is a strong electron acceptor, which means that it can extract electrons from VG or NCG, thus changing the density of the electronic states of the substrate. On the VG, due to defects and reactive edges, a stronger charge transfer to the HAT-CN can be facilitated, which amplifies certain Raman modes through electron resonance effects. This effect could lead to an increase in the intensity of the Raman bands of the HAT-CN but also to a change in the position of the G and 2D bands of the VG. On NCG, the transfer of electric charges is lower due to the more stable character of NCG, but if there are defects or nanosized domains with different Fermi energies, this effect could be visible in the Raman spectra through a change in the relative intensities of the HAT-CN bands, which is not apparent in the spectra presented above.

HAT-CN contains nitrile (-CN) groups, which are extremely sensitive to interactions with the substrate. If significant charge transfer occurs, on VG, the CN groups undergo a slight shift in vibrational frequency due to the change in the electronic density of the molecule, and on NCG, where the interaction is weaker, the position of the CN bands remains relatively stable, but their intensity may be affected by the degree of organization of the molecules on the surface.

### 3.3. FTIR Analysis for Devices Functionalized with TTF on NCG and VG Surfaces

**Figure 6:**
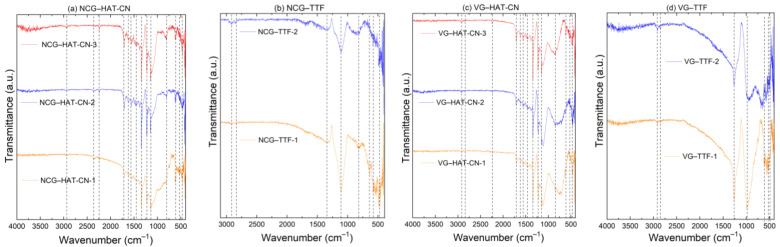
FTIR spectra of GFET devices: (**a**) NCG–HAT-CN; (**b**) NCG–TTF; (**c**) VG–HAT-CN; (**d**) VG–TTF. The labels 1, 2, and 3 refer to individual GFET structures (NCG and VG) obtained on the same Si wafer but functionalized with the specific molecules (TTF and HAT-CN).

The characteristic bands are evidenced in the FTIR spectra of different based samples (Figure 6), and their assignment is as follows: (i) the peaks at 2920, 2849 cm^−1^ (NCG) and 2917, 2849 cm^−1^ (VG) are associated with C–H stretching vibrations, characteristic of aliphatic fragments of TTF or adsorbed methylene groups; (ii) the bands around 1359 cm^−1^ (NCG) and 1262 cm^−1^ (VG) correspond to C–S stretching vibrations, confirming the presence of thiol groups in TTF; (iii) the peaks at 1111 cm^−1^ (NCG) and 1262 cm^−1^ (VG) are attributed to C=C stretching vibrations, originating from the thiolic core of TTF; (iv) the bands at 805 cm^−1^ (NCG) and 978 cm^−1^ (VG) are characteristic of C–S–C bending vibrations, essential for confirming the TTF structure on the surface; (v) the lower frequency bands (640, 610 cm^−1^, and 600 cm^−1^) are associated with vibrational modes of thiolic rings or interactions between TTF molecules and the carbon substrate.

In terms of confirming the functionalization, (i) the presence of TTF-specific bands on both surfaces (VG and NCG) indicates that the molecule has successfully attached to the substrate; (ii) the significant groups (1111/1262, 805, and 640/610 cm^−1^) confirm the interactions between TTF and the carbon surface, validating the success of the attachment process.

The differences between NCG and VG are as follows: (i) The 1111 cm^−1^ band (NCG) is more clearly defined than the 1262 cm^−1^ band (VG), suggesting a stronger interaction between TTF and NCG; (ii) The low-frequency bands (below 600 cm^−1^) show differences in the binding modes, likely influenced by surface morphology—NCG being more disordered, providing more active sites for attachment compared to the more orderly vertical structure of VG.

The results suggest that functionalization is more efficient on NCG than on VG due to the more porous surface and higher number of reactive sites that favor interactions with TTF.

### 3.4. FTIR Analysis of Devices Functionalized with HAT-CN on NCG and VG Surfaces

The decomposition and interpretation of the spectra by key segments is the following: (i) The characteristic nitrile (C≡N) vibrations at 2244 cm^−1^ (NCG) and 2234 cm^−1^ (VG) confirm the presence of nitrile groups in HAT-CN. The slight difference between the two samples suggests that on VG, the value is closer to that of classical aromatic nitrile compounds, which may indicate a weaker or more ordered interaction of HAT-CN molecules on VG; (ii) The aromatic heterocyclic structure vibrations at 1715 cm^−1^ (NCG) and 1706 cm^−1^ (VG) are attributed to C=O vibrations or deformation modes of the aromatic core in the presence of electron-withdrawing groups; (iii) The regions 1620–1564 cm^−1^ (NCG) and 1621–1554 cm^−1^ (VG) correspond to characteristic C=N and C=C stretching vibrations from the heterocyclic structure of HAT-CN; (iv) The 1451–1337 cm^−1^ bands are specific to C–C and C=N vibrations within the aromatic core; (v) The 1224–1148 cm^−1^ region is assigned to C–H and C–N vibrations, indicating interactions between HAT-CN and the carbon surface; (vi) The 799–846 cm^−1^ range corresponds to deformation vibrations of the aromatic system; (vii) The 610–534 cm^−1^ (NCG) and 610–525 cm^−1^ (VG) bands are essential for confirming the presence of HAT-CN, attributed to structural vibrations of the heterocyclic ring and C≡N bonds.

The HAT-CN molecule deposition is confirmed by the following: (i) The most important bands confirming functionalization are 2244/2234 cm^−1^ (C≡N) and 534/525 cm^−1^, confirming the anchoring of nitrile groups on the carbon surfaces; (ii) On VG, the values are closer to those of classical aromatic nitriles, suggesting a more ordered and weaker interaction with the VG substrate compared to NCG.

The differences between NCG and VG are as follows: (i) The 2244 cm^−1^ band (NCG) vs. 2234 cm^−1^ (VG) suggests a stronger interaction with the substrate in the case of NCG; (ii) The 1715 cm^−1^ (NCG) vs. 1706 cm^−1^ (VG) band may indicate a slight electronic influence of the substrate on the HAT-CN molecule; (iii) The low-frequency bands (534/525 cm^−1^) indicate slightly different anchoring of the molecules due to morphological differences between the two substrate types.

### 3.5. Transfer Characteristics of GFET Devices Functionalized with Organic Semiconductor Molecules

**Figure 7:**
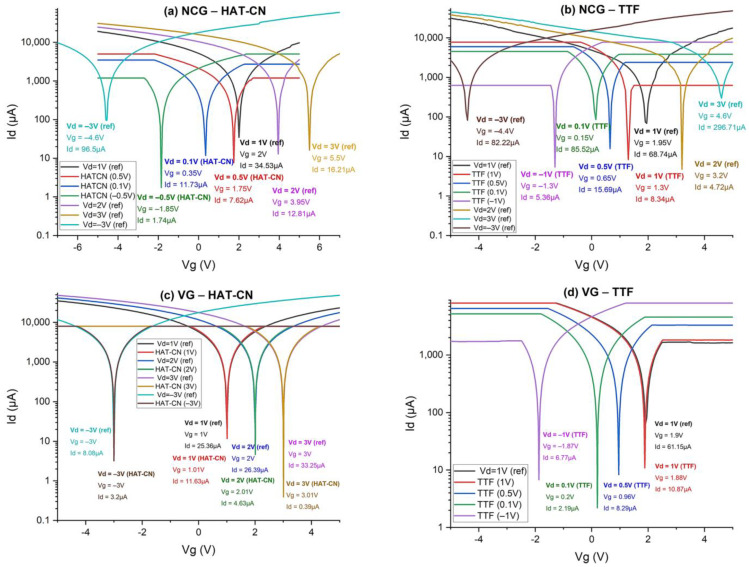
Transfer characteristics of GFETs with a source-drain channel based on NCG and VG functionalized with organic semiconductor molecules TTF and HAT-CN: (**a**) NCG–HAT-CN; (**b**) NCG–TTF; (**c**) VG–HAT-CN; (**d**) VG–TTF.

In Figure 7, it can be seen from the comparison of the transfer characteristics that HAT-CN extracts electrons from the channel, but the interaction is weaker than in the case of TTF, which injects considerably more electrons into the channel than HAT-CN extracts. It is possible that the HAT-CN/channel interface (NCG or VG) is not as efficient, or the molecules have a suboptimal orientation/morphology for efficient charge transfer.

Concerning the two carbonaceous nanomaterials, it is observed that VG has different electronic characteristics compared to planar graphene and NCG, as indicated in our previous works [35,36], which is why the interactions with the organic doping molecules manifest differently due to (i) the topology with numerous edges and defects, (ii) the 3D porous structure, which provides a large active surface area for adsorption and charge transfer, (iii) and the high density of Fermi-level states (N(EF)) around the edges.

The results can be attributed to both the deposition method and the intrinsic material properties used in the experimental settings, as the drop-casting process may lead to substantial morphological artifacts such as non-uniform aggregation and random molecular orientation [37,38]. Moreover, the electrical properties of the graphene-based FETs are clearly influenced by the presence of semiconductor molecules, considering their different types of doping, as the Dirac point shifts according to the specific features of the dopants at the same time as the decrease in current.

The electric measurements suggest a significant impact over the charge transfer properties, owing to the deposition of semiconductor molecules on the GFET devices, as the Dirac point shifts accordingly with the doping type of the deposited organic compounds. However, the recorded current intensities of the GFET structures functionalized with both types of organic semiconductors follows the expected trend in the case of the HAT-CN molecule, as they decrease at each applied potential. The electron-withdrawing properties of HAT-CN reduce the electron density found in the device, thus decreasing the source-drain current.

Discussing the behavior of the GFET structures based on the TTF semiconductor, an increase in source-drain current is expected due to the donor properties of the TTF molecule. Nevertheless, the results shown in Figure 7 also indicate a significant decrease in current after the deposition of TTF on the devices. Considering these facts, the non-uniform aggregation of TTF particles increases the overall resistance of the source-drain channel due to a smaller conductivity of the TTF molecules with its intrinsic organic structure than those of the graphene-derived nanomaterials. The same increase in resistivity also arises in the case of HAT-CN, which presents several areas with aggregates, resulting in a decrease in the registered current.

VG has an excellent ability to accept electrons in its edge regions, and TTF takes advantage of this. HAT-CN is more efficient with planar carbonaceous nanomaterials (such as SLG or NCG), where it can extract electrons uniformly. In VG, HAT-CN may have weaker adsorption or non-optimized orientation and lower charge transfer and be limited to oxidized or defected edge regions. The slight shift of the potential at the Dirac point to the right indicates weak p-type doping, and the slight decrease in current indicates a decrease in conductivity. As such, HAT-CN does not interact as effectively with the three-dimensional structure of VG as TTF does.

The interaction of graphene-based source-drain channels with organic semiconductors such as TTF and HAT-CN significantly modulates the electronic properties of FET devices. These effects are directed by both the molecular nature of the dopants and the morphology of the graphene substrate, either NCG or VG.

TTF, a strong π-electron donor, effectively injects electrons into the graphene channel, inducing a pronounced n-type doping. This is evidenced by a leftward shift of the Dirac point, indicating enhanced channel conductivity. The extended conjugated system of TTF enables strong π–π stacking and orbital overlap with the graphene surface, as confirmed by shifts in characteristic Raman and FTIR bands (e.g., C–S and C=C vibrations). On VG, the charge transfer is more efficient due to the abundance of edge sites and high surface area, while on NCG, TTF binds to more disordered regions, which offer more reactive sites but less controlled molecular orientation.

In contrast, HAT-CN, a strong electron acceptor, exhibits weaker p-type doping behavior. The Dirac point shifts slightly to the right, and a modest current decrease is observed, suggesting electron withdrawal from the channel. Although HAT-CN contains electron-withdrawing cyano groups that support dipole coupling and electrostatic interaction, its performance depends more strongly on the substrate. Raman and FTIR data show that on NCG, HAT-CN binds more uniformly but tends to aggregate densely without strong integration into the graphene lattice. On VG, fewer but better-organized aggregates are observed, with improved interfacial interaction, albeit weaker than with TTF.

Scanning Electron Microscopy (SEM) revealed further substrate-dependent morphological differences. TTF forms small, irregular aggregates on NCG, indicative of a disordered interface, whereas on VG, it deposits more uniformly due to better anchoring on the vertically exposed graphene walls. For HAT-CN, spherical aggregates form on both substrates, but VG supports larger and more organized assemblies, suggesting improved structural directionality and partial intercalation into the 3D graphene structure.

Raman spectroscopy confirmed stronger π–π interactions and charge transfer on VG, with activation of otherwise forbidden modes and increased band intensities (notably D, G, and 2D bands). In contrast, NCG offers a more stable and homogeneous platform that preserves the intrinsic vibrational characteristics of the adsorbed molecules, though with weaker perturbation of the graphene structure.

FTIR analysis supports these findings. Characteristic C–S, C=C, and C–H bands of TTF appeared more clearly on NCG, suggesting stronger thiol interactions due to surface porosity and disorder. However, the more ordered VG surface yielded cleaner spectra and better-aligned molecular layers. For HAT-CN, key C≡N and aromatic ring vibrations confirmed successful functionalization on both substrates, but with stronger interaction signatures on NCG and more ordered alignment on VG.

Therefore, these results demonstrate that graphene morphology critically governs the nature and efficiency of molecular doping and interfacial interaction. VG, with its edge-rich, high-surface-area topology, supports stronger donor–acceptor coupling, enhanced charge transport, and more uniform molecular orientation—particularly advantageous for high-performance sensing and nanoelectronic devices. NCG, while effective in molecule adsorption due to its porosity and reactivity, leads to more disordered interfaces and variable electronic modulation.

## 4. Conclusions

The molecular interaction between each of the two organic semiconductors (HAT-CN and TTF) and the graphene-based materials can be explained by four main physicochemical mechanisms: π–π stacking interactions, charge transfer, dipole–dipole interactions, and van der Waals interactions.

For both semiconductors, π–π stacking interactions are the most important, as both have planar molecules composed of conjugated π-electron systems, like graphene. This structural similarity enables the overlap of π orbitals, leading to strong π–π stacking interactions.

Charge transfer is weaker in the case of HAT-CN but plays a crucial role for TTF, which is a strong electron donor. In the presence of graphene, a charge transfer occurs, in which TTF donates electrons to graphene. This electron transfer can modify the electrical properties of graphene, increasing its conductivity. The charge transfer can be either partial or complete, depending on the experimental conditions.

Dipole–dipole interactions are more significant for HAT-CN, which is a polar molecule due to the presence of nitrile (-CN) groups that exhibit strong dipole moments. These dipole moments can interact with local electric fields in graphene, contributing to the adhesion of HAT-CN molecules on the graphene surface.

TTF symmetry influences the Raman activity on VG and NCG through distortions induced by interaction with the substrate, charge transfer effects, and polarizability change due to thiol functionalization. VG induces more pronounced effects due to its three-dimensional structure and edge defects, while NCG would provide a more stable and predictable interaction.

FTIR confirms the successful functionalization of carbon surfaces with HAT-CN, with stronger anchoring on NCG due to its rougher surface, which offers more interaction sites. In contrast, on VG, the interaction appears more ordered, suggesting a different molecular arrangement on the substrate.

Future research could investigate the application of organic semiconductors like HAT-CN and TTF in graphene-based biosensors. By enhancing the electrical properties of VG and NCG using organic semiconductors, the overall sensitivity of the biosensor could be improved. Future work will also study how TTF and HAT-CN graphene-based biosensors could specifically detect different types of molecules using as receptors single-stranded DNA probes, aptamers, antibodies, or enzymes. Organic semiconductor graphene-based sensors could be further applied in energy and electronic, environmental, and biomedical fields, for energy storage enhancement, development of advanced environmental sensors, and for designing biosensors for early disease detection with remarkable sensitivity and specificity.

## Data Availability

The original contributions presented in this study are included in the article. Further inquiries can be directed to the corresponding author(s).

## Abbreviations

The following abbreviations are used in this manuscript:

NCG	Nano Crystalline Graphite
VG	Vertical Graphene
SLG	Single Layer Graphene
TTF	TetraThiaFulvalene
HAT-CN	HexaAzaTriphenylene-hexaCarboNitrile
FTIR	Fourier Transform Infrared Spectroscopy
FET	Field-Effect Transistor
SEM	Scanning Electron Microscopy
CVD	Chemical Vapor Deposition
PECVD	Plasma-Enhanced Chemical Vapor Phase Deposition
HOMO	Highest Occupied Molecular Orbital
OFET	Organic Field-Effect Transistors
